# Expression of cocaine- and amphetamine-regulated transcript (CART) in hen ovary

**DOI:** 10.1186/s40659-017-0123-x

**Published:** 2017-05-22

**Authors:** Pengfei Li, Xuejing Yu, Jianshan Xie, Xiaolei Yao, Wenzhong Liu, Jianbo Yao, Zhiwei Zhu, Lihua Lyu

**Affiliations:** 1 College of Life Science, Shanxi Agricultural University, Taigu, 030801 Shanxi China; 2 College of Animal Science and Technology, Shanxi Agricultural University, Taigu, 030801 Shanxi China; 3 School of Basic Medical Sciences, Shanxi Medical University, Taiyuan, 030001 Shanxi China; 4 Division of Animal and Nutritional Sciences, West Virginia University, Morgantown, WV 26506 USA

**Keywords:** Hen, Follicles, CART, Theca cell, Granulosa cell

## Abstract

**Background:**

Cocaine- and amphetamine-regulated transcript (CART), discovered initially by via differential display RT-PCR analysis of brains of rats administered cocaine, is expressed mainly in central nervous system or neuronal origin cells, and is involved in a wide range of behaviors, such as regulation of food intake, energy homeostasis, and reproduction. The hens egg-laying rate mainly depends on the developmental status of follicles, expression of CART have not been identified from hen follicles, the regulatory mechanisms of CART biological activities are still unknown. The objective of this study was to characterize the mRNA expression of *CART* in hen follicular granulosa cells and determine CART peptide localization and regulatory role during follicular development.

**Methods:**

Small white follicles (1–2 mm in diameter) were treated for RNA isolation; Small white follicles (1–2 mm in diameter) and large white follicles (4–6 mm in diameter) were treated for immunohistochemical localization and large white follicles (4–6 mm in diameter), small yellow follicles (6–8 mm in diameter), large yellow follicles (9–12 mm in diameter), mature follicles (F5, F4, F3, F2, F1, >12 mm in diameter) were treated for RNA isolation and Real time PCR.

**Results:**

The results showed that full length of the CDS of hen CART was 336 bp encoding a 111 amino acid polypeptide. In the hen ovary, CART peptide was primarily localized to the theca layer, but not all, the oocyte and granulosa layer, with diffused, weaker staining than relative to the theca cell layer. Further, amount of CART mRNA was more (*P* < 0.05) in granulosa cells of 6–8 mm follicles compared with that in granulosa cells of other follicles. However, CART mRNA amount was greater in theca cells of 4–6 mm follicles relative to follicles of other sizes (*P* < 0.05).

**Conclusions:**

Results suggest that CART could play a potential role in developmental regulation of chicken follicles.

## Background

The reproductive performance of hens, especially the egg-laying rate depends mainly on the developmental status of follicles, Follicular development process has the priority features. According to the diameter, follicles can be divided into mature follicles and immature follicles. Mature follicles, the follicles before ovulation (F1, F2, F3, F4, F5$\ldots$), sometimes can be up to 40 mm in diameter. Immature follicles can be divided into small white follicle (SWF, <2 mm), large white follicles (LWF, 3–5 mm), small yellow follicles (SYF, 6–8 mm) and large yellow follicles (LYF, 9–12 mm) [1, 2]. In immature follicles, there is a cuboidal cells layer and basement membrane. As follicle volume increases, granulosa cells begin to proliferate, and theca gradually forms in connective tissue outside the basement membrane, and central egg yolk accumulates. In these small follicles, only a single follicle per day is selected from the cohort of follicles of 6–8 mm in diameter into the pre-ovulatory hierarchy to begin rapid growth and final differentiation. Ovaries were studied and only two types of atresia were identified—non-bursting and bursting. Smaller, non-yolky follicles (<1 mm diameter) showed non-bursting atresia. Atresia in follicles >1 mm diameter was invariably of the bursting type [3]; these two types are related to the developmental stage or size of the follicle that becomes atretic.

CART is an endogenous neuropeptide which is widespread in animals. Kobayashi et al. [4] first discovered *CART* mRNA expressed in cow ovaries, which was localized in the antral follicles oocytes, granulosa and cumulus cells by immunohistochemistry and in situ hybridization. Further research found that when granulosa cells were treated with a certain dose of CART, the generation of E_2_ was inhibited in granulosa cells, the effect depends on the stage of cell differentiation, suggesting that CART could play a crucial role in regulating follicle atresia [5]. Lv et al. [6] showed that *CART* mRNA amounts in subordinate follicles were significantly greater than that in dominant follicles. E_2_ secretion levels decreased by CART injection in early dominant follicle, and *CYP19A1* (Cytochrome P450, family 19, subfamily A, polypeptide 1) mRNA expression levels reduced in granulosa cells, demonstrated that CART could cause bovine follicular atresia. For mammals, E_2_ was synthesized and secreted by granulosa cells, but for poultry, E_2_ was mainly synthesized and secreted by theca cells [7]. Jochnson et al. [8] found estradiol was feedback by paracrine pathway to control the secretion of progesterone in granulosa cells, directly affected on the follicle, involved in regulating ovulation. With estrogen increasing, the sensitivity of follicles to hormone increased, play a decisive role to the formation of the dominant follicle, low estrogen synthesis follicles become blocked, and eventually apoptosis [1, 9, 10]. Recently, our laboratory has confirmed that CART plays a crucial role in inhibiting the proliferation of granulosa cells and the secretion of E_2_ in cattle, pig and sheep by cell culture in vitro [6, 11, 12]. CART acts as a potent inhibitor of promoting granulosa cells apoptosis by down-regulating FSH-induced cAMP amount, E_2_ accumulation and aromatase mRNA levels [13, 14]. It is unknown if CART is expressed in the follicles of laying hens. The relationship between the CART expression and follicular development in different stages of laying hens remains to be determined. Thus, in this study, we hypothesized that CART is expressed in laying hens’ follicle. Immunohistochemical localization and qRT-PCR were performed to detect the *CART* mRNA expression in granulosa cells and theca cells in different sizes of hens’ follicles.

## Methods

### Animals

All animal experiments in this study were carried out in strict accordance with the recommendations in the *Guide* for the Care and Use of Laboratory Animals of the National Institutes of Health.

Six healthy hens were selected, and ovaries were collected and follicles in different diameters were treated for RNA isolation, tissue fixing and the separation of granulosa cells and theca cells, respectively.

### RNA isolation and cDNA synthesis

Total RNA was isolated from the small white follicles of hens using Trizol (Takara, Dalian, China) according to the manufacturer’s instructions. Isolated RNA was dissolved in RNase-free sterile water treated with 0.1% (vol/vol) diethylpyrocarbonate. Before cDNA synthesis, 2.5 μg total RNA were incubated with 2.0 μL 5 × gDNA eraser buffer and 1.0 μL gDNA eraser (Takara, Dalian, China) at 42 °C for 2 min to remove genomic DNA. Then 4 μL of 5 × PrimeScript^®^ Buffer 2,1 μL of RT Primer Mix,1 μL of PrimeScript^®^ RT Enzyme Mix I (Takara, Dalian, China), and RNA free water up to 20 μL. The cDNA was synthesized at 37 °C for 15 min and 85 °C for 5 s, transferred to a sterile screw-cap micro-centrifuge tube, and stored at −20 °C for further use.

### Cloning of *CART* cDNA

The chicken *CART* gene sequence is not available in the National Center for Biotechnology Information (NCBI) GenBank database. Thus, DNAMAN software was used to identify the similarity of bovine, human being, rat and porcine *CART* cDNA sequences, a pair of primers were designed for PCR amplification of the hen *CART* cDNA sequence (Table 1). The identity of *CART* amplicons generated via RT-PCR was determined by agarose gel electrophoresis analysis. Total RNA from small white follicles from adult ovary were reverse transcribed, and respective cDNAs were amplified by PCR. The cycler program used consisted of 35 cycles at 94 °C for 30 s, 57 °C for 30 s, and 72 °C for 1 min, with a final extension at 72 °C for 5 min. The amplified cDNA encoding partial CART was ligated into the pMD 19-T Vector (TAKARA, Dalian, China). Plasmids containing inserts of interest were then subjected to fluorescent dye terminator sequencing via Beijing Genomics Institute (BGI, China).

**Table 1:** Primer sequences used in this study

Primer name	Sequence (5′ to 3′)	Tm (°C)	Size (bp)
RT-PCR			
CART-F	AGCGCGGCTCGGCGGGATTCGGCAGC	58.7	396
CART-R	GGGCGGACGTGCACCGCGCCGGTGCC	58.7	
qRT-PCR			
CART-R-F	GAGAAGGAGCTGATCGAGGC	60.0	88
CART-R-R	CCTGCCCGAACTTCTTTTCG	59.5	
β-actin-F	AATGGCTCCGGTATGTGCAA	60.0	112
β-actin-R	GGCCCATACCAACCATCACA	60.0	

F, sense primers; R, antisense primers

### Immunohistochemical localization of the CART peptide

Small white follicles (1–2 mm in diameter) and large white follicles (4–6 mm in diameter) were collected at a local abattoir from ovaries of three different hens. Samples were placed in a plastic tissue cassette, fixed in Bouin’s buffer for 20–24 h, washed in 70% (vol/vol) alcohol until the yellow color is gone. Then tissues were dehydrated and embedded in paraffin. Immunohistochemical localization of the CART peptide was performed using previously described procedures [15] using rabbit anti rat CART (55–102) polyclonal antisera (Phoenix Pharmaceuticals, Inc., Belmont, CA) at a 1:2000 dilution. Parallel controls were used, including sections incubated with a similar dilution of normal rabbit serum or rabbit anti-CART serum that had been pre-incubated overnight at 4 °C with 10 g/mL rat CART (55–102) peptide (American Peptide Co., Sunnyvale, CA). Ten serial sections from each sample were examined.

### The isolation of granulosa cells and theca cells

Ovaries were collected at a local abattoir based on the follicular diameter: the first largest, second largest, third largest, fourth largest, fifth largest follicles (F1, F2, F3, F4, F5) and the 9–12, 6–8, 4–6 mm follicles (n = 5) were dissected, and washed with 0.9% saline. Follicular fluid was aspirated from each follicle, stored in −20 °C refrigerator. Follicle shell was cut so that it was almost bisected, but not completely cut through. The inner wall of the follicle was gently scraped to remove the granulosa cells (scrape slightly only one time so as not to get theca cells). The follicle shell was then removed from the watch glass and placed in a petri dish with media for theca isolation. Medium containing granulosa cells was transferred to a sterile 15 mL tube on ice containing 2 mL medium using pipette. The watch glass with remaining cells was rinsed with medium which was then transferred into a 15 mL tube. The theca cells were then isolated under a stereomicroscope. Using 2 pairs of fine forceps to peel the theca interna (yellow) from the theca externa (white), starting at the edges of the cut flaps. The isolated granulosa cells and theca cells were frozen in liquid nitrogen for 5 s, then stored in −80 °C refrigerator before RNA extraction.

### Quantitative real-time PCR

Real-time RT-PCR was used to quantify amounts of *CART* mRNA in granulosa cells and theca cells. Total RNA from both types of cells were used for analysis (n = 5 each). Synthesis of cDNA was performed as described above. Primers were designed using the Primer premier 5.0 program (http://www.premierbiosoft.com) with chicken *CART* nucleotide sequence obtained above. The PCR mixture contained 100 ng cDNA, 10 μL SYBR^®^ Premix Ex TaqII (TAKARA, Dalian, China), ROX Reference Dye II 0.4 μL, 8 pM forward and reverse primer (CART-R-F, CART-R-R, Table 1) in a total reaction volume of 20 μL. As an internal control, the amount of *β*-*actin* mRNA in each sample was quantified using chicken *β*-*actin* gene specific primers (primers are listed in Table 1). Reactions were performed in duplicate for each sample in an ABI PRISM 7000 Sequence Detection System (Applied Bio-systems). The thermal cycler program consisted of 45 cycles of 95 °C for 5 s and 60 °C for 30 s. The amounts of *CART* and *β*-*actin* mRNA in each sample were determined by comparison of cycle threshold for each sample with respective *β*-*actin* mRNA’s. The relative mRNA expression level of AGTR2 was calculated using the comparative 2^−ΔΔCT^ method [16].

### Statistical analysis

The amount of *CART* mRNA and *β*-*actin* mRNA in follicles was analyzed using the general linear model procedure of SPASS (version 17.0, USA). Amounts of *CART* mRNA were normalized relative to *β*-*actin* mRNA, and data were log-transformed before analyses. Data are shown as mean ± SE.

## Results

### Cloning and sequence analysis of hens *CART* CDS

**Fig. 1:**
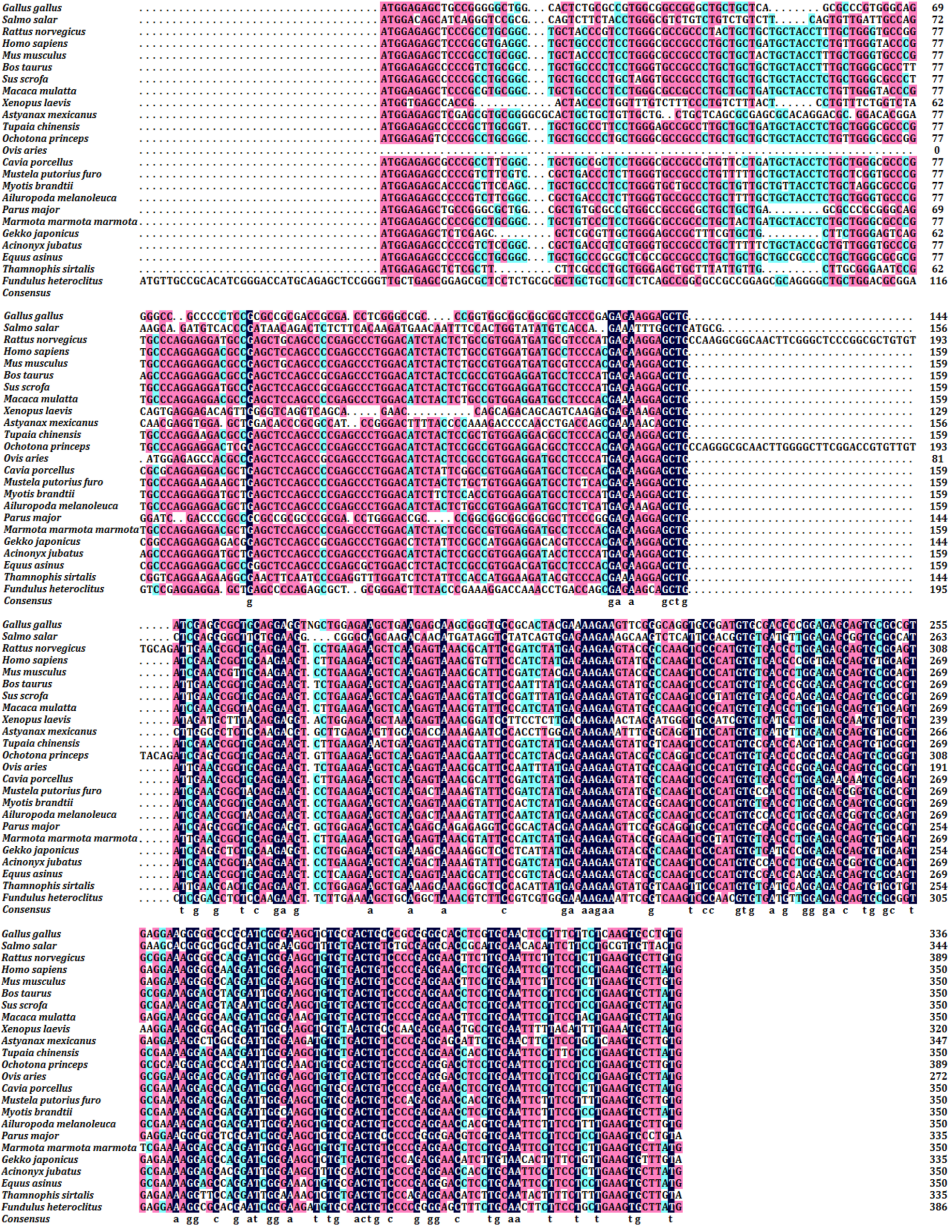
Multiple alignment of nucleotide sequences of hen ovarian follicular *CART* with other species. Identical/similar sequences were highlighted in *black*/*pink*, *white* and *blue* background in corresponding species. *Hyphens* indicated gaps in order to optimize the alignment. The *last line* indicated consensus nucleotide of different species

**Fig. 2:**
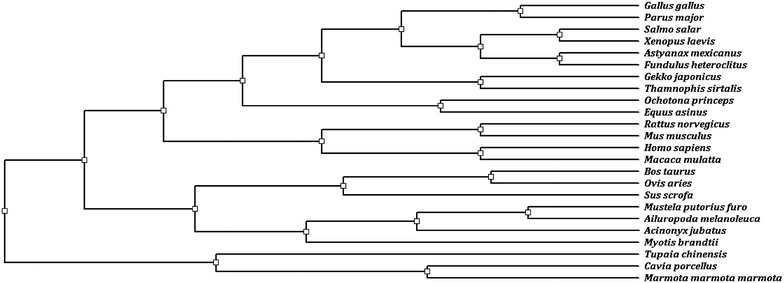
Homological analysis of hen relative to other organisms by phylogenetic analysis. Sequences alignment of CART nucleotide sequence was processed by a Observed Divergency method in DNAMAN program

A complete hen *CART* CDS was obtained by PCR, 336 bp in length. The nucleotide sequence of hen *CART* displayed 90.8% similarity to Parus major and 75.3–79.2% of shared identity with others species (Fig. 1). In order to examine the relationship of hen *CART* and its counterparts in various other organisms, a phylogenetic tree of CART peptides from hen and other species was constructed (Fig. 2). The topology of the tree demonstrated that there were six groups in the entire alignment of animals including mammalia, verschiedene fischgerichte, primates, reptiles, birds and rodents. The phylogenetic analysis showed that hen CART peptide was closely related to Parus Major CART.

### Intra-follicular expression of CART peptide

**Fig. 3:**
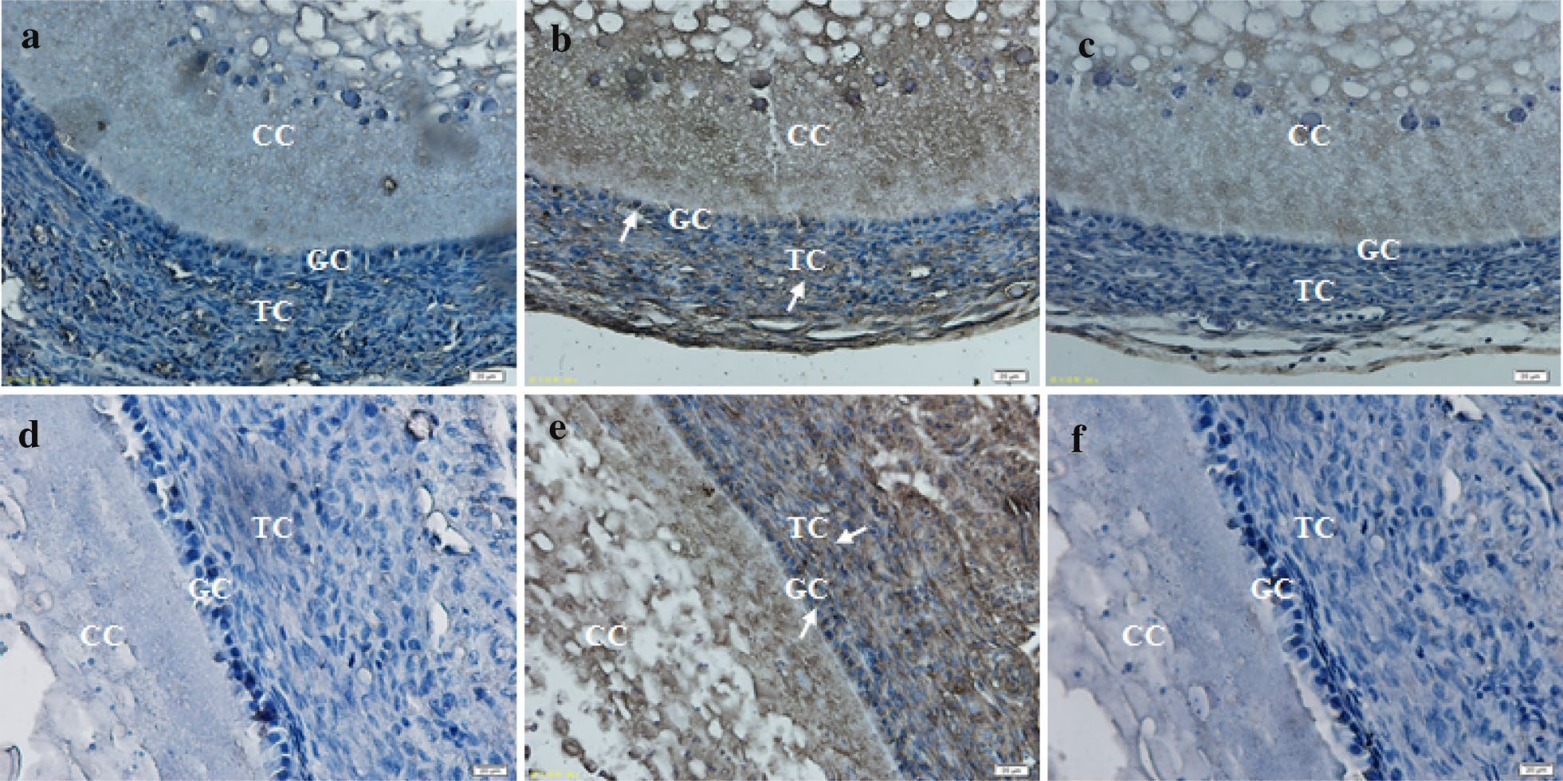
Expression of CART peptide in chicken ovaries by immunohistochemistry (×400). **a**, **d** Micrograph of adjacent sections (to that depicted in **b**, **e**) were incubated with normal rabbit serum. **b**, **e** representative micrograph of section through stroma of adult hen ovary follicles were incubated with rabbit anti-CART serum. **c**, **f**, Micrograph of adjacent section (to that depicted in **b**, **e**) were incubated with rabbit anti-CART serum pre-absorbed with an excess of CART peptide. **a**–**c**, *small white* follicles (1–2 mm in diameter); **d**–**f** *large white* follicles (4–6 mm in diameter). *GC* granulosa cell layer, *TC* theca cell layer, *CC* Cumulus cells. **a**–**f**: Magnification 400; *scale bar* 20 µm

The intra-ovarian localization of the CART peptide was determined using immunohistochemistry (Fig. 3). Prominent CART immune-reactivity was localized to the theca layer. CART immune-reactivity was also localized to the granulosa layer, but with diffused, weaker staining than the theca cell layer. Significant immune-reactivity in the granulosa cells, cumulus cells, and theca cells were not detected when adjacent sections were incubated with normal rabbit serum or when the CART antiserum was pre-absorbed with excess CART peptide.

### Differential expression of *CART* mRNA

**Fig. 4:**
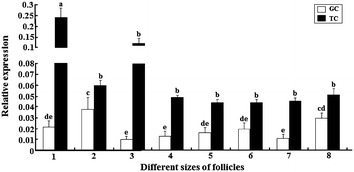
Relative expression of *CART* mRNA in granulosa cells (*White column*) and theca cells (*Black column*) of the follicles in different sizes. Note: 1: 4–6 mm follicles (*large white* follicles); 2: 6–8 mm follicles (small yellow follicles); 3: 9–12 mm follicles (large yellow follicles); 4: F5; 5: F4; 6: F3; 7: F2; 8: F1 follicles (mature follicles, F1>F2>F3>F4>F5, and all the five follicles are >12 mm in diameter). (*Superscript small letters* indicate significantly different, values with the *same letters* were not significantly different and values with the *different letters* were significantly different at the level of 0.05)

To further determine the association of *CART* expression with stages of follicular growth and development, the expression of *CART* mRNA in granulosa cells and theca cells of different size follicles (n = 5 each) was determined. As expected, amount of *CART* mRNA was more (*P* < 0.05) in granulosa cells of 6–8 mm follicles compared with that in granulosa cells of other follicles except F1. The *CART* mRNA amounts were greater in F1 granulosa cells than that in 9–12 mm follicles, F5 and F2 follicles (*P* < 0.05) (Fig. 4, White column). However, *CART* mRNA amount was greater in theca cells of 4–6 mm follicles relative to follicles of other sizes (*P* < 0.05) (Fig. 4, Black column). In every follicle of different size, the expression of *CART* mRNA was higher in theca cells than in the same size follicle’s granulosa cells (P < 0.05) (Fig. 4).

## Discussion

Poultry follicular development is a highly dynamic physiological process, which is coordinated by a variety of hormones and cytokines remote autocrine, paracrine and autocrine and other means to promote granulosa cell proliferation and differentiation, endometrial cells appear and oocyte maturation, direct or indirect control of follicular development occurs and until ovulation [9, 17]. *CART* mRNA expressed in hypothalamus of multiple mammalian [18], and *CART* mRNA has been detected in follicles of cattle [11, 14], pig [12] and sheep [19]. We found CART mRNA and protein were expressed in follicles of hen too.

Evidence indicates CART is a novel intraovarian regulator of follicular development in numerous species. The mature CART is a potent negative regulator of FSH-induced [11, 14] and IGF1-induced [20] E_2_ production in vitro and can inhibit follicular E_2_ production in vivo [6]. In cattle, follicular fluid CART concentrations in healthy follicles decrease after dominant follicle selection, and CART mRNA is lower in healthy vs atretic follicles collected before and early after initiation of follicle dominance, suggestive of a regulatory role in the selection process [6]. The inhibitory actions of CART on FSH signaling and E_2_ production depend on the Go/i-subclass of inhibitory G proteins and are linked to multiple components of the FSH signal transduction pathway, resulting in reduced *CYP19A1* mRNA and E_2_ production [11, 14]. CYP19A1 is the steroidogenic enzymes responsible for androgen synthesis and the aromatization of androgens to estrogens [21]. In immature chicken ovaries, exogenous FSH induces steroidogenesis by increasing *CYP19A1* mRNA expression and subsequent progesterone synthesis [22]. qRT-PCR results showed that *CART* mRNA expression level was significantly higher within the largest pre-ovulatory follicle (F1) granulosa cells than that in those follicles with >12 mm in diameter (F5 and F2), this is consistent with Tilly’s results [23], indicating the negative relationship between CART expression levels and estrogen amounts. *CART* mRNA were greatly expressed in theca cell layer of the follicles (4–6 mm), resulting in an increasing of CART peptide expression of small yellow follicles (6–8 mm) in the next stage. Previous research suggested that theca cell layers of follicles (4–6 mm) were thinner than that in other follicles, and the theca cells layers are the main sources of estrogen and testosterone [23–25], inhibiting the synthesis and secretion of E_2_. It further validates that small yellow follicle is selected and then develops into a preovulatory follicles [26, 27]. Preovulatory follicles rarely become atretic under normal physiological conditions. Follicle recruitment into the preovulatory hierarchy is accompanied by the first evidence of FSH-induced cAMP accumulation [23] and increased basal levels of LH receptor (LHR) mRNA [28] within the rapidly differentiating granulosa cell layer.

CART treatment of ovine granulosa cells had pronounced inhibitory effects on FSH-induced E_2_ production and blocked the FSH-induced increase in granulosa cells numbers observed over 7 day culture period [29], and results of studies demonstrate a similar yet distinct response of ovine granulosa cells to CART treatment as observed for the bovine system. Furthermore, recent studies support a prominent requirement of Wnt signaling for mediating stimulatory effects of FSH on E_2_ production and granulosa cell proliferation [30]. Investigation of direct effects of CART stimulation on Wnt signaling linked to E_2_ production and proliferation of hen granulosa cells is a focus of future studies.

In summary, results of present studies demonstrated that CART is expressed in granulosa and thecal layers of hen follicles, differential expression of CART based on follicular size and cellular layer in hen ovary, and the follicles (6–8 mm in diameter) at this time is the key turning point to continue to develop into the dominant follicle or atresia, results support a potential role for CART in regulation of follicular development in the hen. However, it is important to note that follicular dynamics and regulation in hen are distinct from that noted for cattle and ovine. It is acknowledged that study design was not optimal due to limited sample collection and test maneuverability,because the big follicles (>6 mm in diameter) could not dehydration for immunohistochemical localization, and small white follicles (1–2 mm in diameter) could not isolation of granulosa cells and theca cells for qRT-PCR. Despite such limitations, results have significantly enhanced understanding of hen ovary potential differences in CART expression associated with follicular development that are foundational to further study in the future. Hence, further study of CART potential Wnt signaling linked to regulation of atresia are necessary to dissect its potential species-specific role in regulation of follicular development.

## Conclusion

*CART* mRNA and CART peptide were expressed in granulosa cells and theca cells of follicles in different sizes, this could affect steroidogenesis to further influence the hen follicular development, suggesting CART plays a potential role in developmental regulation of chicken follicles.
